# Exploring Mean Platelet Volume and Platelet Count Recovery in Dengue Patients: Findings From an Observational Retrospective Clinical Study

**DOI:** 10.7759/cureus.65553

**Published:** 2024-07-28

**Authors:** Sidharth S Pattnaik, Urvin Patil, Lovy Aggarwal, Shubhransu Patro, Purusottam Mishra, Ambika Mohanty

**Affiliations:** 1 Internal Medicine, Kalinga Institute of Medical Sciences, Bhubaneswar, IND

**Keywords:** thrombocytopenia, inflammation, platelet count recovery, mean platelet volume, dengue

## Abstract

Introduction

Mean platelet volume (MPV) measures platelet size in the blood, which is important because dengue fever often leads to low platelet counts, especially during the critical phase. However, predicting when a patient's platelet count will recover is challenging due to the lack of clinical data. MPV may offer a solution as it tends to rise when platelet counts fall, suggesting a possible link to bone marrow activity. This study aims to understand how MPV changes during the three phases of dengue fever and how it relates to platelet count recovery. Successful results could provide valuable markers for clinicians, helping improve patient care and management.

Material

The study was carried out in the Department of Medicine, Kalinga Institute of Medical Sciences (KIMS), Bhubaneswar, Odisha, India. The patients who were admitted with dengue fever/dengue haemorrhagic fever in the month of July 2023 were analysed.

Observations

A total of 130 patients were analysed. The average (avg) MPV on day one was 10.85 ± 1.56, on day three was 10 ± 1.48, and on day five was 9.80 ± 1.30. The avg. total platelet on day one was 119476.92 ± 78,107.78, on day three was 119000 ± 59962.52, and on day five was 169200 ± 100839.84. The correlation between MPV and platelet on day one was r= -0.22, p=0.011, which was statistically significant; on day three was r= -0.32, p=0.0001, which was statistically significant, and on day five was r= -0.30, p= 0.0004, which was statistically significant.

Conclusion

These findings suggest that as dengue fever progresses, MPV tends to increase as platelet counts decline. This information can be beneficial in clinical practice as it highlights the potential utility of MPV as a predictive marker for platelet recovery, aiding healthcare providers in the timely management of dengue patients to mitigate bleeding risks.

## Introduction

Dengue fever, stemming from the dengue virus, presents a formidable global health concern, especially prevalent in tropical and subtropical regions [1]. With an estimated 390 million infections occurring annually, the disease burden of dengue is substantial, leading to considerable morbidity and mortality [2]. Platelets, crucial components of the hemostatic system, play a pivotal role in the pathogenesis of dengue fever. In the early phase of the infection, there occurs a rapid drop in platelet levels due to bone marrow suppression and peripheral destruction, resulting in thrombocytopenia, a defining trait of dengue fever. [3].

A multitude of investigations has centred on platelet counts as a gauge of the severity and advancement of illness in individuals affected by dengue. However, emerging studies propose that mean platelet volume (MPV), a measure of both platelet size and activation, could serve as an indicative factor for predicting the recovery trajectory in dengue patients [4]. While platelet count primarily signifies the abundance of platelets circulating within the bloodstream, MPV offers a deeper understanding of platelet function and turnover [5].

The hypothesis driving this investigation is that alterations in MPV during the course of dengue infection could serve as a predictive biomarker for patient recovery. Specifically, we postulate that changes in MPV levels may negatively correlate with the resolution of dengue symptoms and the restoration of platelet homeostasis. By monitoring MPV dynamics alongside platelet counts, clinicians may gain valuable prognostic information to guide patient management and therapeutic interventions [6].

Several mechanisms underpinning the potential relationship between MPV and dengue recovery warrant exploration. Firstly, alterations in MPV may reflect ongoing platelet activation and turnover in response to virus-induced endothelial dysfunction and immune-mediated processes. Secondly, changes in MPV may signify the transition from acute inflammation to resolution, reflecting the balance between pro-inflammatory and anti-inflammatory cytokines in dengue pathophysiology [5,7].

Moreover, studies have indicated the association between MPV and clinical outcomes in various infectious and inflammatory conditions like urinary tract infection (UTI), septicaemia, and SARS-CoV2, highlighting its potential utility as a prognostic marker [8]. For instance, elevated MPV has been linked to disease severity and poor prognosis in sepsis, indicating its role as a marker of systemic inflammation and coagulopathy [9].

This study aims to elucidate the rationale behind investigating MPV as a predictor of recovery in dengue patients, drawing upon existing literature and clinical observations. By examining the evidence supporting the role of MPV in dengue pathogenesis and recovery, this study seeks to contribute to the growing body of knowledge aimed at improving prognostication and patient outcomes in dengue fever.

## Materials and methods

The study was conducted at the Department of Medicine, Kalinga Institute of Medical Sciences (KIMS), Bhubaneswar, Odisha, India, and was designed as a retrospective observational study. Consecutive patients admitted to the Department of Medicine at KIMS with a diagnosis of either dengue fever or dengue hemorrhagic fever during July 2023 were included. Exclusion criteria comprised diagnosed cases of any malignancy, patients with quantitative and qualitative platelet disorders, those on anti-platelet drugs, and patients with a diagnosis of hemolytic anaemia.

Sample size calculation

For a small correlation where the correlation coefficient is 0.2, the formula for calculating sample size is: n= {(Z1−α/2)2+(Z1−β)2 }/2 +3, where Z1−α/2=1.96 Z_{1-\alpha/2} = 1.96Z1−α/2=1.96 (for 95% confidence)and Z1−β=0.84 Z_{1-\beta} = 0.84Z1−β=0.84 (for 80% power), which gives n=109.24+3 or n≈112.24 approx. So, for a small correlation (ρ≈0.2\rho \approx. 0.2ρ≈0.2) with 95% confidence and 80% power, a sample size of approximately 112 patients would be needed. A total of 130 patients were kept as sample size including 5% dropouts.

Demographic data such as age, gender, and residential area, alongside clinical data including presenting symptoms, duration of illness, and past medical history, were collected from patient records. Laboratory investigations included complete blood count, platelet count, MPV, and serological tests for dengue virus (NS1 antigen and IgM/IgG antibodies), which were conducted using the hospital's facilities. Descriptive statistics were employed to summarize demographic characteristics and laboratory findings. Categorical variables were presented as frequencies and percentages, while continuous variables were expressed as means with standard deviations or medians with interquartile ranges, as appropriate. Data skewness was analyzed, and depending on the distribution, appropriate statistical tests, such as the Spearman correlation, were applied.

To assess the distribution characteristics of the dataset, we initially performed skewness analysis. Given the critical role of skewness in determining the appropriate statistical tests, we employed several methods to evaluate skewness.

Visual inspection

Histograms and box plots were generated for a visual assessment of the distribution symmetry.

Descriptive statistics

Skewness Coefficient

The skewness coefficient was calculated using the e1071::skewness function in R. The skewness coefficient quantifies the asymmetry of the data distribution.

Normality tests

Shapiro-Wilk Test

We conducted the Shapiro-Wilk test to assess the normality of the data. A significant p-value (typically < 0.05) suggests a deviation from normality, indicating skewness.

Distribution of Platelet Parameters

The Shapiro-Wilk test for normality analysis revealed that the total platelet count data followed a normal distribution, while the MPV data did not exhibit a normal distribution. As a result, non-parametric tests of significance were employed for further analysis.

Data analysis was conducted using the statistical programming language R (version 4.3.2) (R Foundation, Vienna, Austria).

This study was performed in accordance with the principles of the Declaration of Helsinki, with ethical approval obtained from the Institutional Ethics Committee of Kalinga Institute of Medical Sciences (KIIT/KIMS/IEC/1763/2024) prior to the study's commencement. Patient confidentiality was rigorously maintained, and data were anonymized during analysis to protect patient privacy.

## Results

A total of 130 patients were analysed from July 2023. The average (avg.) MPV on day one was 10.85 ±1.56, on day three was 10 ± 1.48, and on day five was 9.80±1.30. The avg. total platelet on day one was 119476.92 ± 78,107.78, on day three was 119000 ± 59962.52, and on day five was 169200 ± 100839.84. The correlation between MPV and platelet on day one was r= -0.22, p=0.011, which was statistically significant, on day three was r= -0.32, p=0.0001, which was statistically significant, and on day five was r= -0.30, p= 0.0004, which was statistically significant.

Summary statistics

For MPV, the median values on day one, day three, and day five were 10.8, 10, and 9.80, respectively, with corresponding interquartile ranges (IQRs) of 2.45, 2.35, and 2.1.

Regarding total platelet counts, the mean values on day one, day three, and day five were 119,476.92, 119,000, and 169,200, respectively, with SDs of 78,107.78, 59,962.52, and 100,839.84.

Correlation analysis

Spearman correlation tests revealed significant negative correlations between MPV and total platelet counts on day one (rho = -0.220849, p = 0.01157), day three (rho = -0.324442, p = 0.000166), and day five (rho = -0.3045147, p = 0.0004275). These findings suggest that as total platelet counts increase, MPV tends to decrease, and vice versa (Table 1).

**Table 1:** Correlation between platelet counts and MPV on days one, three, and five of dengue fever MPV: mean platelet volume

Correlated parameters	p-value	Correlation coefficient (rho)	Confidence interval (95%)
Day one MPV ~ day one total platelets	0.011	-0.2208	-0.3815, -0.0556
Day three MPV ~ day three total platelets	0.0001	-0.324	-0.4655, -0.1646
Day five MPV ~ day five total platelets	0.0004	-0.304	-0.4487, -0.1406

Regression analysis

Regression analyses were conducted to further explore the relationship between MPV and total platelet counts on each day using the Generalized Linear Model (GLM) from R language (4.3.2).

A GLM is an extension of the traditional linear regression model. It allows for the dependent variable to have a non-normal distribution and relates it to the independent variables via a link function. In this case, the relationship between MPV and total platelet counts over days one, three, and five was explored using a GLM.

The identity link function was used, which meant that the model assumed a linear relationship between the dependent and independent variables. The formula used was η=Xβ\eta = X\betaη=Xβ, where η\etaη is the linear predictor (mean of the dependent variable), XXX is the matrix of independent variables, and β\betaβ is the vector of coefficients.

**Figure 1:**
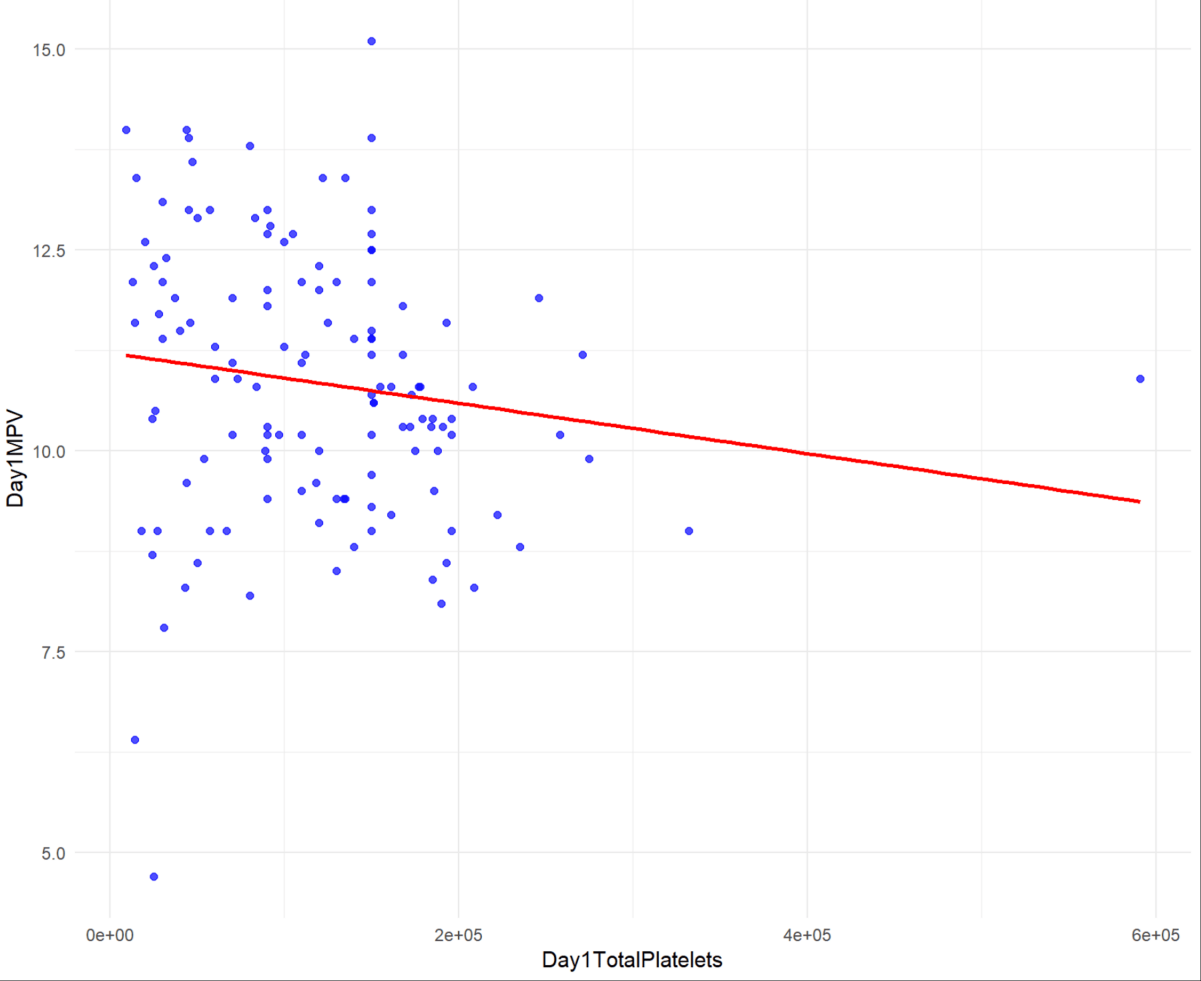
Scatter plot and regression line of day one total platelet counts vs day one MPV MPV: mean platelet volume

**Figure 2:**
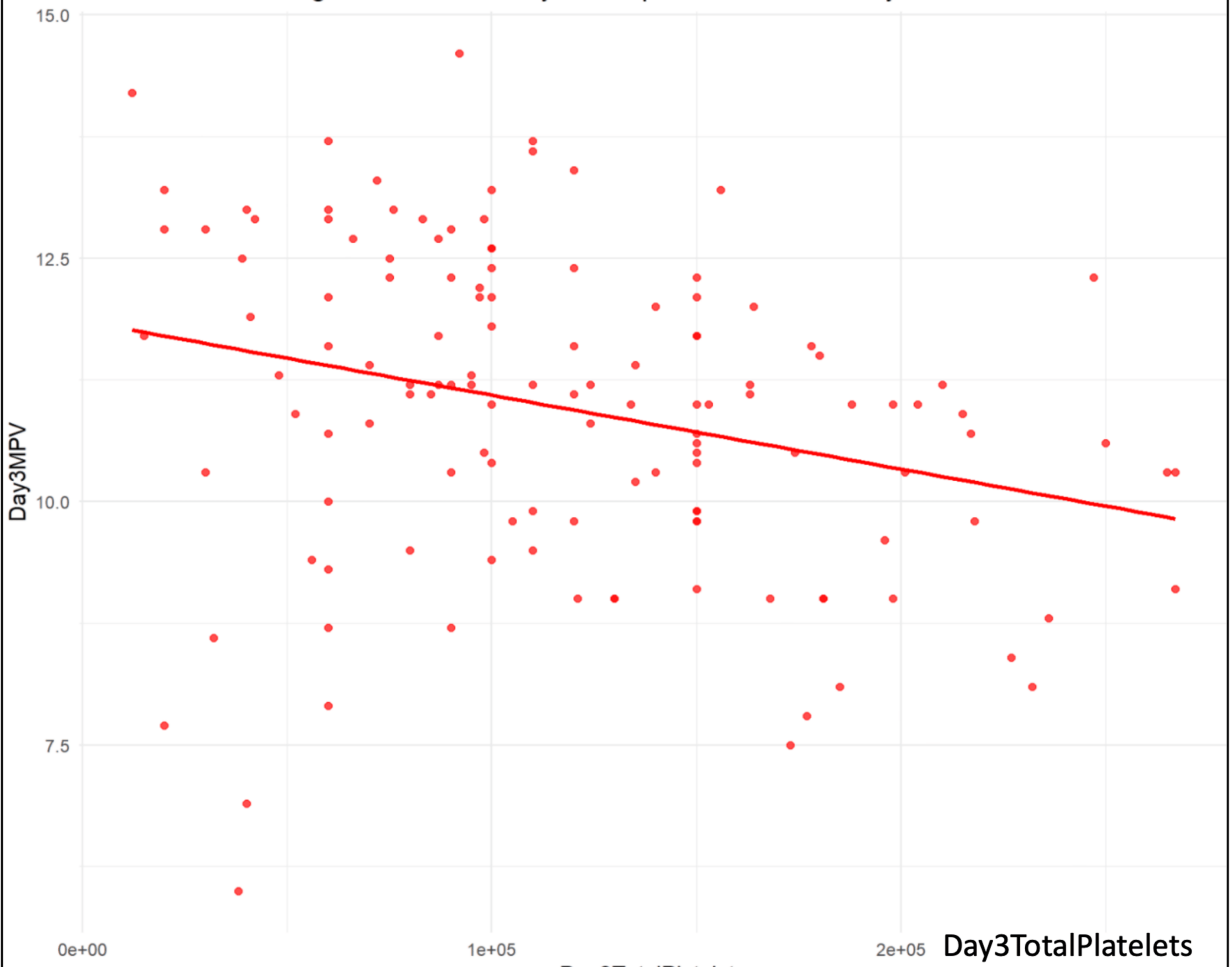
Scatter plot and regression line of day three platelet counts vs day one MPV MPV: mean platelet volume

**Figure 3:**
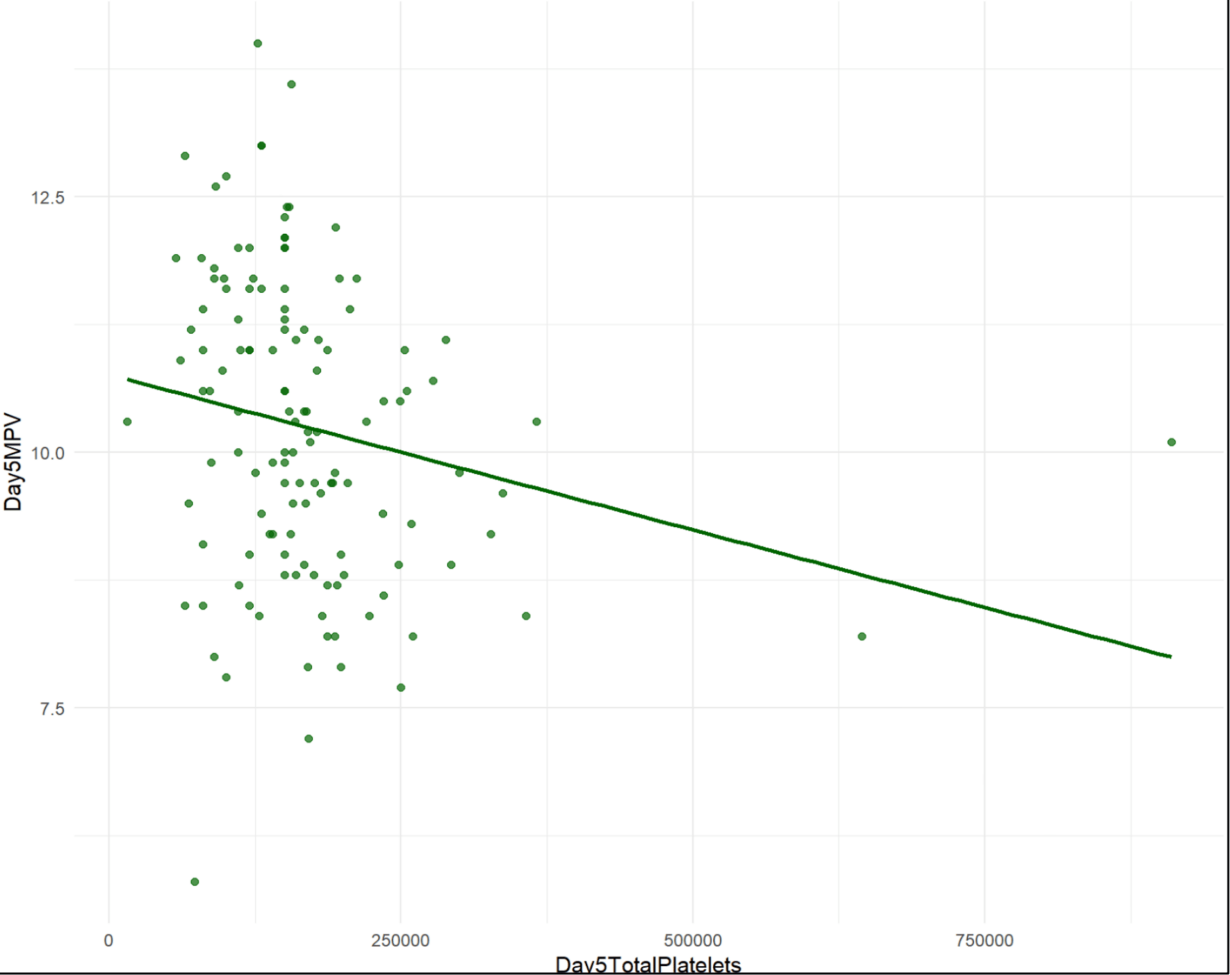
Scatter plot and regression line of day five platelet counts vs day one MPV MPV: mean platelet volume

The results indicated a significant negative association between MPV and total platelet counts on day three (β = -7.596e-06, p = 0.00128) and day five (β = -3.036e-06, p = 0.01718), while the association on day one showed a trend towards significance (β = -3.134e-06, p = 0.101) (Table 2) (Figures 1-3).

**Table 2:** Regression analysis parameters between MPV and total platelet counts on days one, three, and five MPV: mean platelet volume

Parameter	Residual (median)	Coefficient	p-value	Confidence interval (95%)
MPV ~ total platelet day one	0.04	-3.134e-06	0.1	(-7.03e-06, 7.59e-07)
MPV ~ total platelet day three	0.16	-7.596e-06	0.001	(-1.15e-05, -4.11e-06)
MPV ~ total platelet day five	0.002	-3.036e-06	0.017	(-5.52e-06, -5.48e-07)

## Discussion

The results of this study demonstrate a consistent negative correlation between MPV and total platelet counts across multiple time points in patients with dengue fever or dengue hemorrhagic fever. These findings suggest a potential relationship between platelet size and platelet count dynamics during the course of the disease, which may have implications for understanding disease pathogenesis and guiding clinical management strategies.

The findings of this study regarding the relationship between MPV and total platelet counts in patients with dengue fever or dengue hemorrhagic fever align with previous research in the field. Several similar studies have reported a negative correlation between MPV and platelet counts, suggesting that changes in platelet size may be associated with alterations in platelet production or consumption during dengue infection.

For instance, a study by Srichaikul et al. [10] conducted in Thailand provided valuable insights into platelet function during the acute phase of dengue hemorrhagic fever. Their findings of a significant negative correlation between MPV and platelet counts underscored the potential utility of platelet indices as prognostic markers for disease severity. Similarly, the study by Asha et al. [11] identified platelet indices including MPV as a simple risk predictor for severe dengue, highlighting the clinical relevance of platelet parameters in risk stratification and management decisions. This study was conducted with 250 dengue patients at Thrissur, Kerala, India, representing the regional trends of dengue fever and platelet indices. Hence we have additional supportive evidence that MPV can be a useful prognostic marker irrespective of geographical location [11].

Additionally, the study by Shahila M et al. [12] further corroborated the negative correlation between MPV and platelet counts observed in our study. Their investigation into MPV as a predictive factor for platelet recovery in dengue fever provided valuable insights into the dynamics of platelet indices during the course of the disease. Together, these studies support the generalizability of our findings across different geographic regions and patient populations, enhancing the robustness of the evidence base regarding platelet parameters in dengue infection.

These consistent findings across different parts of Southeast Asia and different patient populations support the notion that alterations in platelet parameters, such as MPV, may serve as valuable biomarkers for disease severity and prognosis in dengue infection. The negative correlation observed in our study suggests that as platelet counts decrease, there is a tendency for platelets to become larger, potentially reflecting increased platelet turnover or activation in response to the viral infection.

The implications of these findings extend beyond diagnostic considerations to potential therapeutic implications. For example, interventions aimed at modulating platelet function or production could be explored as adjunctive therapies for managing dengue-associated thrombocytopenia and preventing disease progression. Additionally, the use of MPV as a prognostic marker for identifying patients at a higher risk of developing severe dengue complications warrants further investigation.

Further research is warranted to elucidate the underlying mechanisms driving these associations and to evaluate the utility of MPV as a prognostic marker or therapeutic target in dengue management. Additionally, future studies should consider potential confounding factors and explore the generalizability of these findings to broader patient populations.

Limitations

Despite the strengths of our study, including a comprehensive analysis of platelet parameters over multiple time points, there are limitations that should be acknowledged. The observational and not-controlled nature of the study and reliance on secondary data may introduce biases or confounding variables that were not accounted for in the analysis. Additionally, the sample size and single-centre design may limit the generalizability of the findings to broader patient populations.

## Conclusions

In conclusion, the negative correlation between MPV and platelet counts observed in this study adds to the growing body of evidence supporting the utility of platelet parameters as biomarkers for dengue severity and prognosis. Future research should focus on elucidating the underlying mechanisms driving these associations and evaluating the clinical utility of MPV as a predictive or therapeutic target in dengue management.
